# Supplementary material for: Synthesis, Characterization and Photocatalytic Activity of CoFe2O4/Fe2O3 Dispersed in Mesoporous KIT-6
Source: Nanomaterials (Basel). 2022 Oct 12;12(20):3566. doi: 10.3390/nano12203566 (PMC9611405; doi:10.3390/nano12203566)
Supplement: Supplementary file 1 [file nanomaterials-12-03566-s001.zip › nanomaterials-1914665-supplementary.pdf]

nanomaterials

Supplementary Materials

Synthesis, Characterization and Photocatalytic Activity of $CoFe_2O_4/Fe_2O_3$ Dispersed in Mesoporous KIT-6

Johnatan de Oliveira Soares [1], Wesley Eulálio Cabral Cavalcanti [1], Marco Antonio Morales Torres [2], Sibele Berenice Castella Pergher [1], Fernando José Volpi Eusébio De Oliveira [1] and Tiago Pinheiro Braga [1,*]

[1] Instituto de Química Laboratório de Peneiras Moleculares (LABPEMOL), Instituto de Química, Universidade Federal do Rio Grande do Norte, Natal 59078-970, RN, Brazil
[2] Departamento de Física Teórica e Experimental, Universidade Federal do Rio Grande do Norte, Natal 59078-970, RN, Brazil
* Correspondence: tiago.braga@ufrn.br; Tel.: +55-8433422323 (code:108)

Citation: Soares, J.d.O.; Cavalcanti, W.E.C.; Torres, M.A.M.; Pergher, S.B.C.; De Oliveira, F.J.V.E.; Braga, T.P. Synthesis, Characterization and Photocatalytic Activity of $CoFe_2O_4/Fe_2O_3$ Dispersed in Mesoporous KIT-6. *Nanomaterials* **2022**, *12*, 3566. https://doi.org/10.3390/nano12203566

Received: 29 August 2022
Accepted: 6 October 2022
Published: 12 October 2022

Publisher's Note: MDPI stays neutral with regard to jurisdictional claims in published maps and institutional affiliations.

Copyright: © 2022 by the authors. Licensee MDPI, Basel, Switzerland. This article is an open access article distributed under the terms and conditions of the Creative Commons Attribution (CC BY) license (https://creativecommons.org/licenses/by/4.0/).

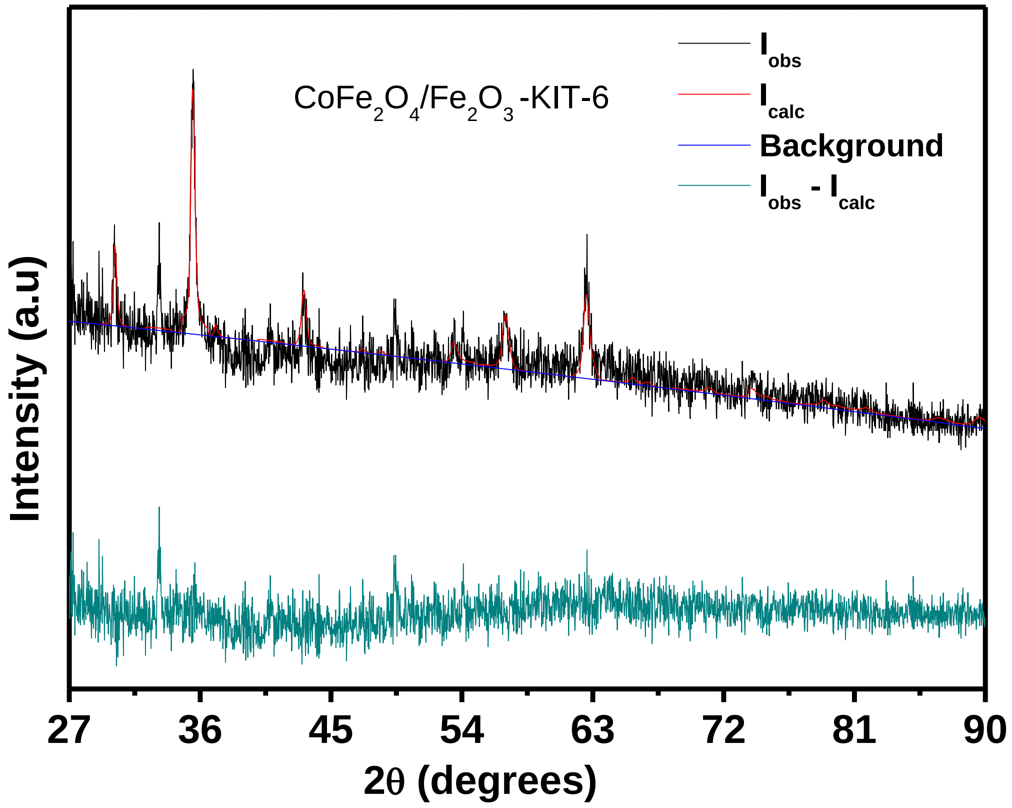

Figure S1. Rietveld refinement for the $CoFe_2O_4/Fe_2O_3$-KIT-6 solid.

Table S1. Mesoporous structure parameters: Interplanar distance($D_{(211)}$) and mesoporous structure parameter for cubic structure (a_{0Cubic}).

**Sample**	**$D_{(211)}$**	**a_{0cubic}**
KIT-6	9.014	22.079
$CoFe_2O_4/Fe_2O_4$-KIT-6	8.429	20.646

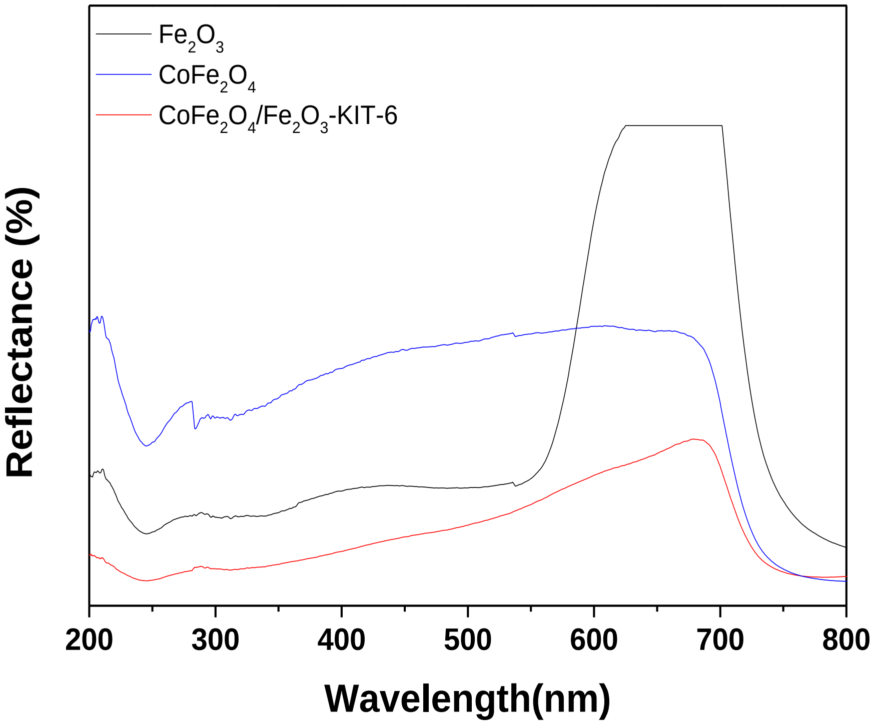

Figure S2. Diffuse reflectance spectra (DRS) of $CoFe_2O_4/Fe_2O_3$-KIT-6 solid, pure Fe_2O_3 and pure $CoFe_2O_4$.

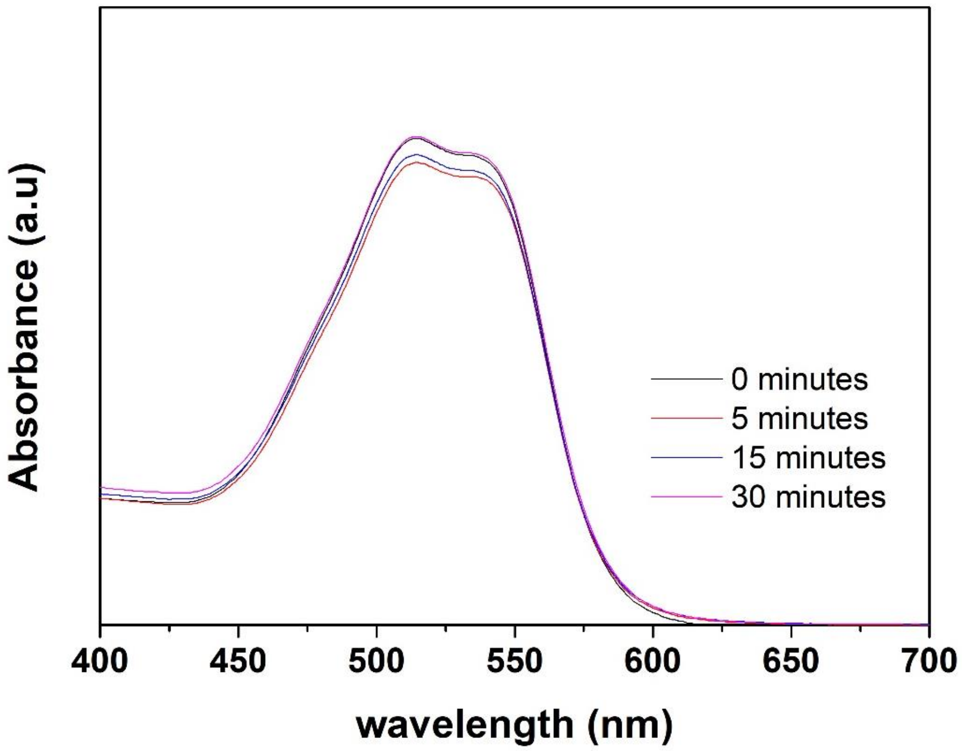

Figure S3. Dye adsorption in 30 min in the absence of light using $CoFe_2O_4/Fe_2O_3$-KIT-6 photocatalyst.

The anatase phase (TiO_2) was added to the $CoFe_2O_4/Fe_2O_3$-KIT-6 catalyst to verify if there was an improvement in the degradation process compared to the solid proposed in the study. The XRD of the $TiO_2/CoFe_2O_4/Fe_2O_3$-KIT-6 photocatalyst is described in Figure S4. The titanium oxide impregnation process was carried out successfully, forming anatase, in the proportion of 10% by mass, Figure S4. It was not possible to identify the Fe_2O_3 phase, probably due to its low concentration and the increase in the number of phases present in the solid.

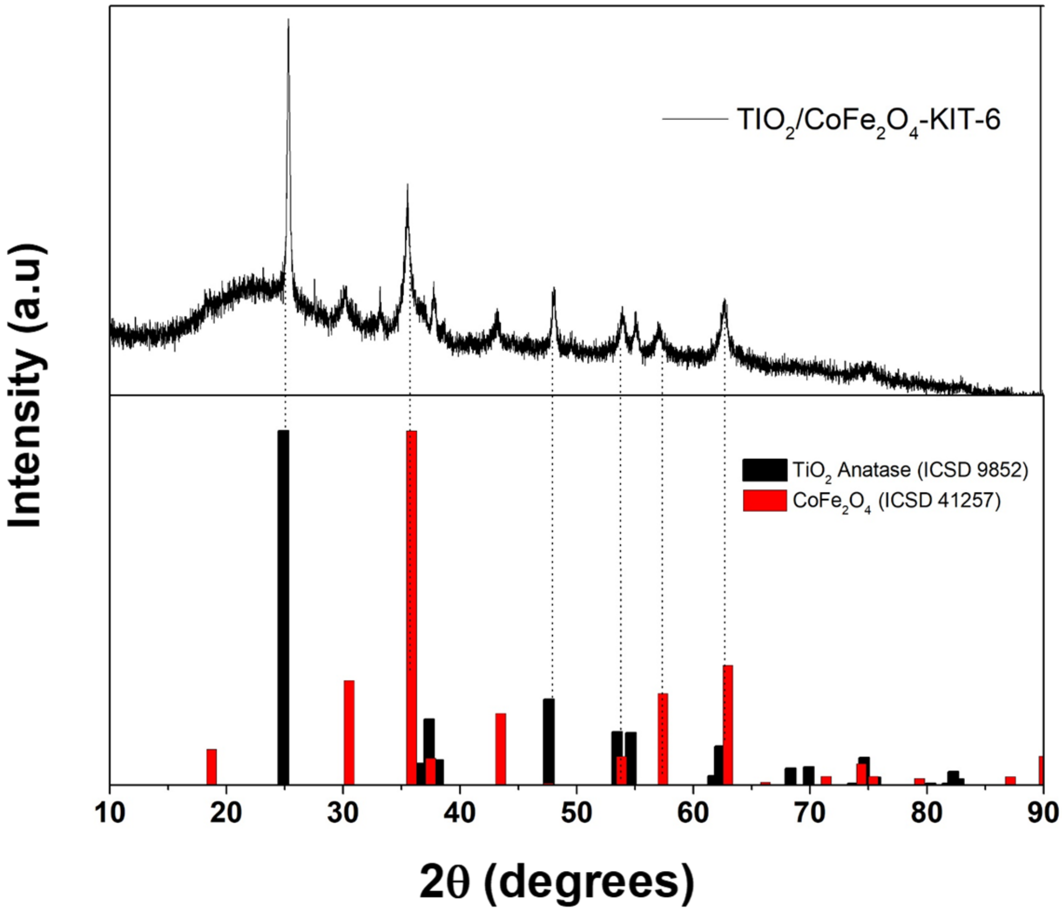

Figure S4. XRD result for the TiO2/CoFe2O4/Fe2O3-KIT-6 photocatalyst.

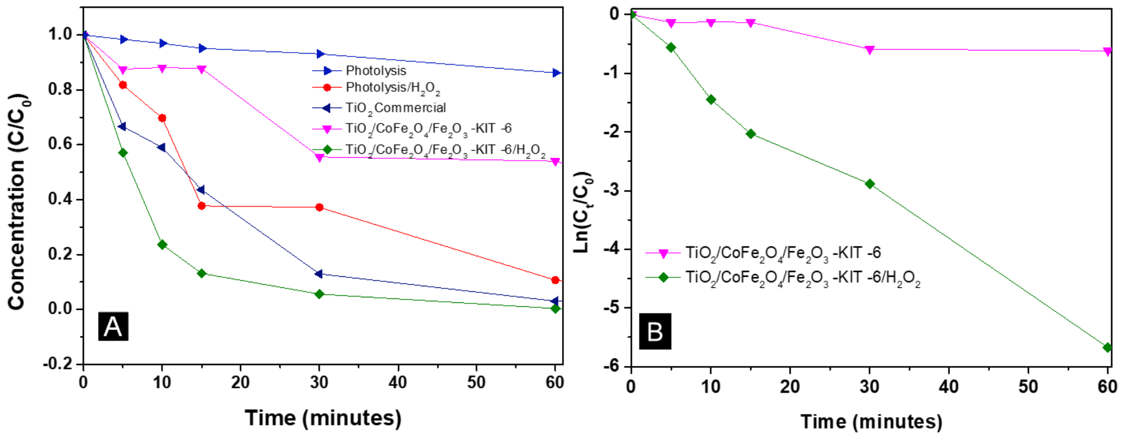

Figure S5. Tests for the TiO2/CoFe2O4/Fe2O3–KIT-6 catalyst. (A) degradation of the URRD dye and (B) kinetic parameter of the reaction.

Table S2. Degradation rate and kinetic parameters for systems with $TiO_2/CoFe_2O_4/Fe_2O_3$–KIT-6 for 1h of reaction.

Sample	Degradation rate (%)	R^2	k` (min-1)	$t_{1/2}$(min)
$TiO_2/CoFe_2O_4/Fe_2O_3$–KIT-6 without H_2O_2	46	0.96	0.00975	71.1
$TiO_2/CoFe_2O_4/Fe_2O_3$–KIT-6 with H_2O_2	99	0.97	0.09229	7.51

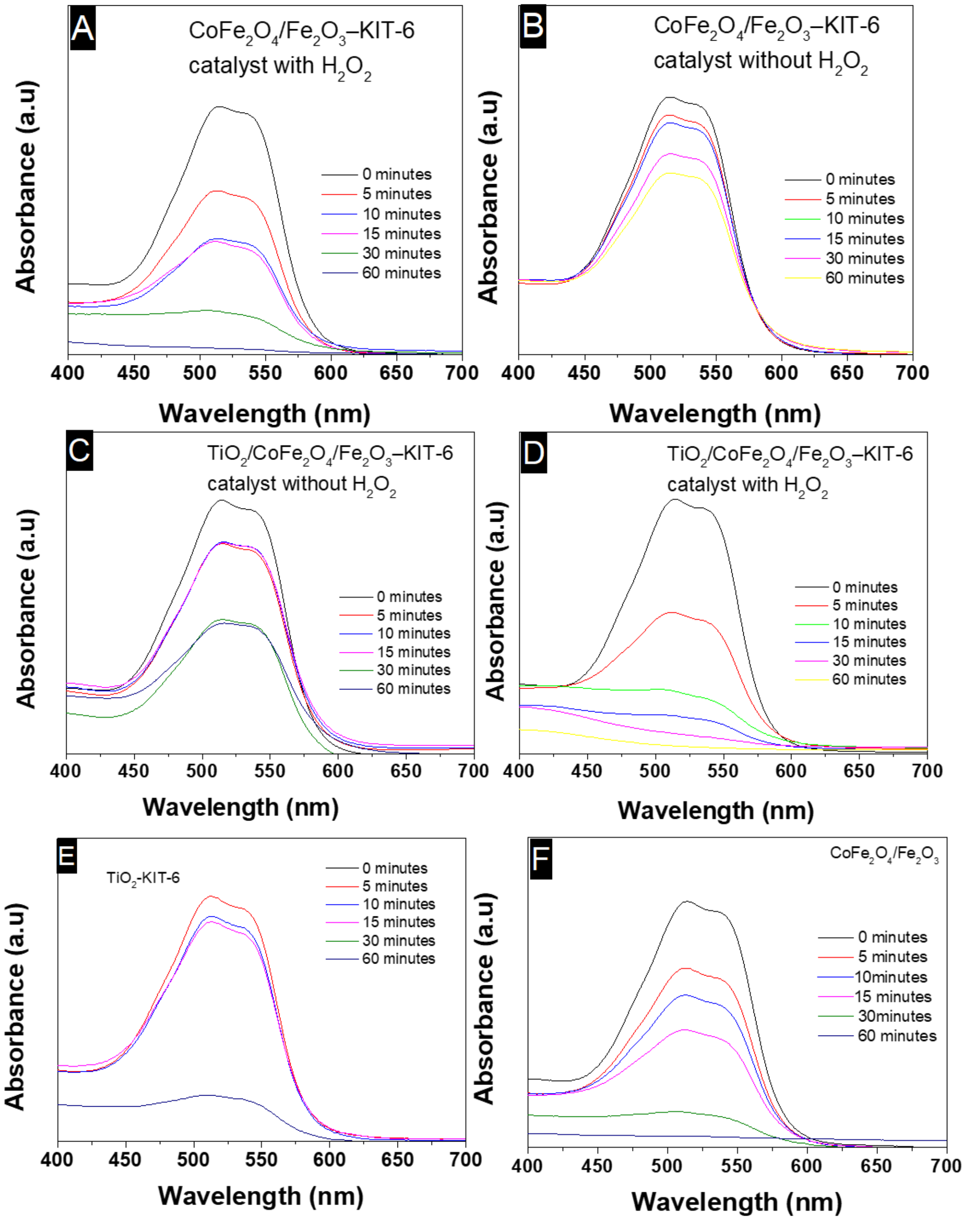

Figure S6. Degradation of the URRD dye. (A) $CoFe_2O_4/Fe_2O_3$–KIT-6 catalyst with H_2O_2; (B) $CoFe_2O_4/Fe_2O_3$–KIT-6 catalyst without H_2O_2; (C) $TIO_2/CoFe_2O_4/Fe_2O_3$–KIT-6 catalyst without H_2O_2; (D) $TIO_2/CoFe_2O_4/Fe_2O_3$–KIT-6 catalyst with H_2O_2.; (E) TiO_2/KIT-6 catalyst with H_2O_2; (F) $CoFe_2O_4/Fe_2O_3$ solid with H_2O_2.

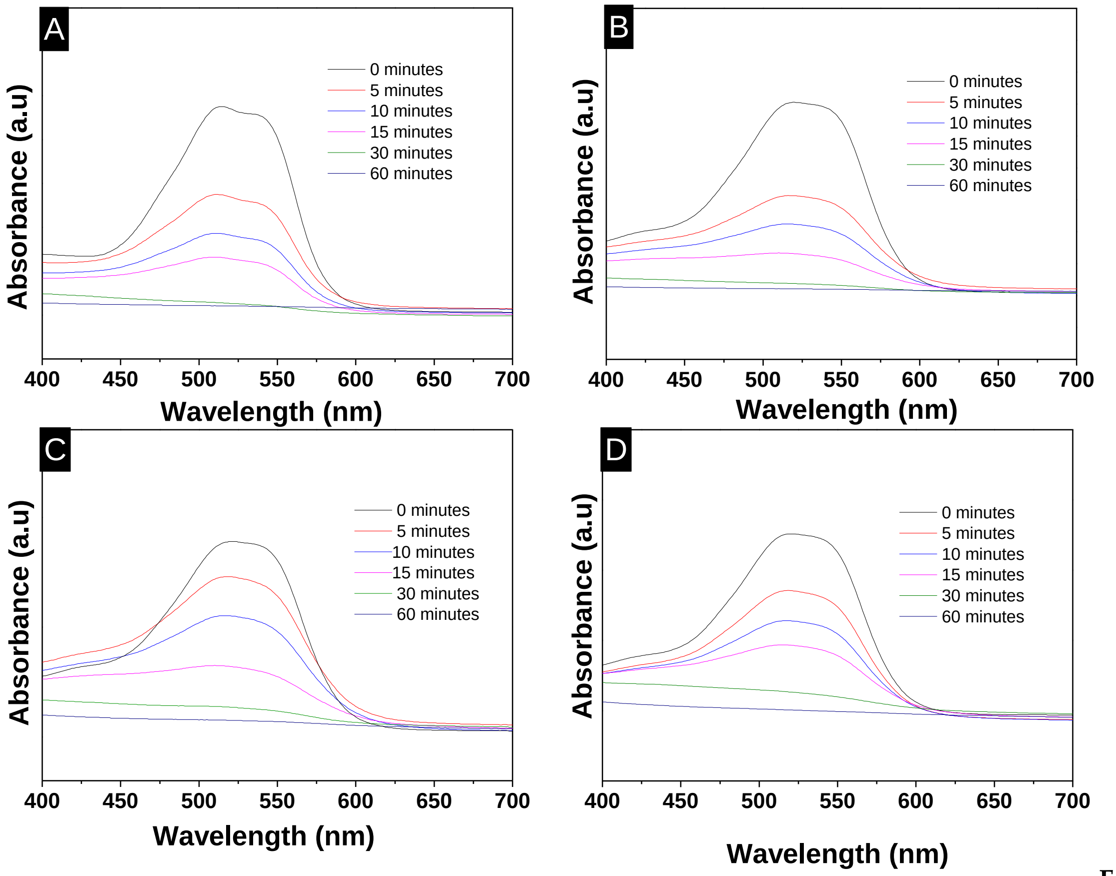

Figure S7. Photocatalyst reuse. (A) first cycle; (B) second cycle; (C) Third cycle; (D) fourth cycle.

Table S3. Possible by-products for the photocatalytic degradation of the URRD dye in 15 min of reaction.

Major by-products	Molecular Weigth (g mol^{-1})	Structure	% Similarity with NIST	Major íons
1-Methyl - 3 -phenylindole	207	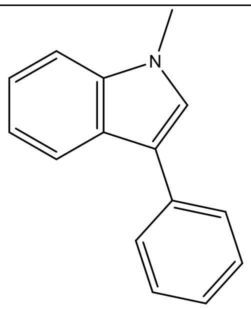	75	96,133, 191, 207
2,4-Dimethylbenzo[h]quinoline	207	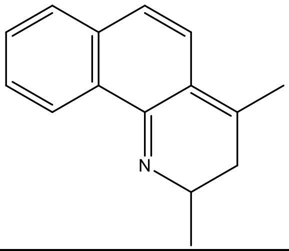	74	96,133, 191, 207
